# Platelets and Defective N-Glycosylation

**DOI:** 10.3390/ijms21165630

**Published:** 2020-08-06

**Authors:** Elmina Mammadova-Bach, Jaak Jaeken, Thomas Gudermann, Attila Braun

**Affiliations:** 1 Division of Nephrology, Department of Medicine IV, Hospital of the Ludwig Maximilian University of Munich, 80336 Munich, Germany; 2 Center for Metabolic Diseases, KU Leuven, 3000 Leuven, Belgium; jaak.jaeken@kuleuven.be; 3 Walther-Straub-Institute for Pharmacology and Toxicology, Ludwig Maximilian University, 80336 Munich, Germany; thomas.gudermann@lrz.uni-muenchen.de

**Keywords:** N-glycans, platelets, thrombosis, hemostasis, megakaryopoiesis, congenital disorders of N-glycosylation

## Abstract

N-glycans are covalently linked to an asparagine residue in a simple acceptor sequence of proteins, called a sequon. This modification is important for protein folding, enhancing thermodynamic stability, and decreasing abnormal protein aggregation within the endoplasmic reticulum (ER), for the lifetime and for the subcellular localization of proteins besides other functions. Hypoglycosylation is the hallmark of a group of rare genetic diseases called congenital disorders of glycosylation (CDG). These diseases are due to defects in glycan synthesis, processing, and attachment to proteins and lipids, thereby modifying signaling functions and metabolic pathways. Defects in N-glycosylation and O-glycosylation constitute the largest CDG groups. Clotting and anticlotting factor defects as well as a tendency to thrombosis or bleeding have been described in CDG patients. However, N-glycosylation of platelet proteins has been poorly investigated in CDG. In this review, we highlight normal and deficient N-glycosylation of platelet-derived molecules and discuss the involvement of platelets in the congenital disorders of N-glycosylation.

## 1. Introduction

**Figure 1:**
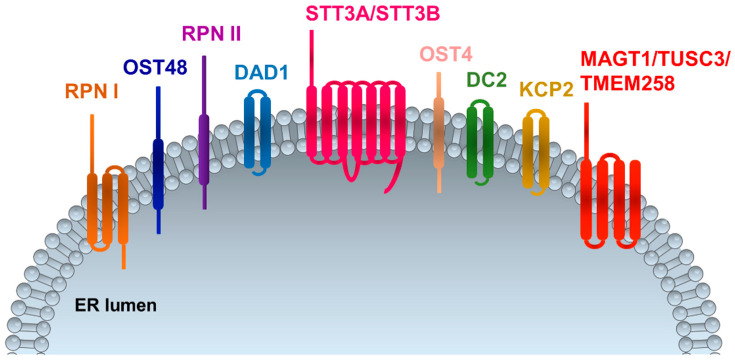
Oligosaccharyltransferase (OST) complex subunits in mammalians. RPNI and RPNII: ribophorin I and II; OST4 and OST48: oligosaccharyltransferase 4 and 48; DAD1: defender against cell death 1; STT3A and STT3B: oligosaccharyltransferase complex catalytic subunit A and B; DC2: oligosaccharyltransferase complex non-catalytic subunit; KCP2: keratinocyte-associated protein 2; MAGT1: magnesium transporter 1; TUSC3: tumor suppressor candidate 3; TMEM258: transmembrane protein 258. ER: endoplasmic reticulum.

N (asparagine)-linked protein glycosylation is a conserved process of protein modification that eukaryotic cells use for protein folding, assembly, and trafficking. Attachment of N-linked glycans (sugar “trees”) to polypeptides takes place in the endoplasmic reticulum, where the oligosaccharyltransferase (OST) complex catalyzes the transfer of a high mannose oligosaccharide onto asparagine residues within the primary protein sequence of Asn–X–Ser or Asn–X–Thr (NXS/T), where X is any amino acid except proline [1,2]. The OST complex in humans is a protein complex composed of several subunits, including STT3 oligosaccharyltransferase complex catalytic subunit A (STT3A), subunit B (STT3B), tumor suppressor candidate 3 (TUSC3), magnesium transporter 1 (MAGT1), transmembrane protein 258 (TMEM258), keratinocyte-associated protein 2 (KCP2), ribophorins (RPN1-2), defender against cell death 1 (DAD1), oligosaccharyltransferase complex subunit 4 and 48 (OST4 and OST48), and oligosaccharyltransferase complex non-catalytic subunit (DC2) proteins [2,3,4,5] (Figure 1). So far, two OST complexes have been identified in mammalians with catalytic subunits STT3A or STT3B associated with different non-catalytic subunits [2]. They couple oligosaccharides to create a specific sugar tree on the protein surface [2]. RPNs link ribosomes to the ER surface during protein translation and present substrates to the catalytic center and additionally function as a chaperone that recognizes misfolded proteins [5,6]. STT3B is associated with the subunits MAGT1 and TUSC3, while STT3A is exclusively associated with KCP2 [7,8]. Although 90% of N-glycosylation is generated through the STT3A complex, the STT3B complex frequently modifies residues with a disulfide-bonded cysteine within the amino acid motif of NCS/T, thus indicating that the function of MAGT1/TUSC3 could be a thiol-disulfide oxidoreductase within the sequon, required for efficient N-glycosylation. MAGT1 is also associated with calreticulin, further supporting the function of the STT3B complex in the process of N-glycosylation [7].

**Figure 2:**
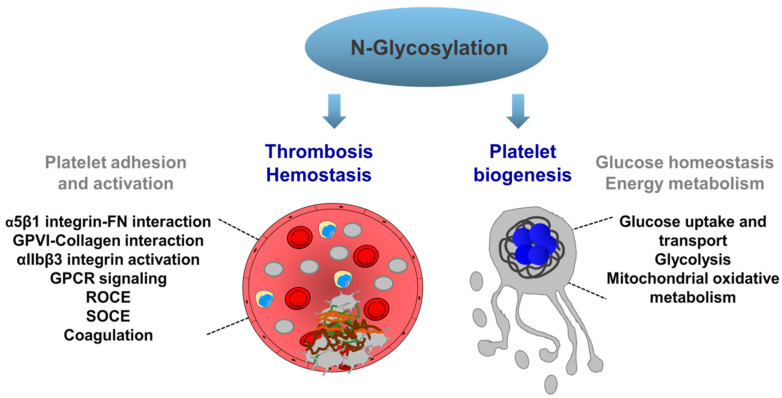
N-glycosylation-mediated mechanisms regulating pathophysiological functions in platelets and megakaryocytes. N-glycosylation of platelet receptors may influence platelet adhesion, activation through regulation receptor–ligand interactions, receptor processing, and signaling activation. N-glycosylation is also involved in calcium homeostasis, ROCE and SOCE in platelets. In addition, N-glycosylation of platelet granule proteins and coagulation factors may regulate important steps in thrombosis and hemostasis, such as granule release, platelet procoagulancy and blood clotting. Glucose uptake and transport, glycolysis mitochondrial oxidative metabolism are regulated by proteins undergoing N-glycosylation, which influence glucose homeostasis, and energy metabolism and consequently platelet biogenesis. FN: fibronectin; GPVI: Glycoprotein VI; GPCR: G-protein-coupled receptors; ROCE: receptor-operated Ca^2+^ entry; SOCE: store-operated Ca^2+^ entry.

Defective N-glycosylation has been observed under pathological conditions, such as cancer, inflammation, diabetes, and myocardial infarction, thus underscoring the importance of understanding the functional significance of N-glycans [9]. N-glycans in mammalians play an important role in many biological processes, such as cell adhesion, and migration by modulating functions of many cell adhesion molecules, including various glycoproteins and integrins [10]. Although several components of the OST complex exist in megakaryocytes and platelets, the functional role of the protein complex under normal and disease conditions has not been investigated. In this review, we focus on normal and deficient N-glycosylation of platelet-derived molecules regulating several functions of platelets and megakaryocytes in thrombosis, hemostasis, and platelet biogenesis (Figure 2). We also discuss platelet involvement in the congenital disorders of N-glycosylation (N-CDG).

## 2. Regulation of Platelet Receptor Function by N-Glycosylation

Platelets are small anucleated fragments derived from megakaryocytes in bone marrow sinusoids, circulating in the blood. Upon vascular injury, subendothelial matrix proteins, such as collagen are exposed to the blood flow, anchoring von-Willebrand-Factor (vWF) and initiating platelet glycoprotein (GP)Ibα–vWF interaction and subsequent GPVI–collagen interaction, a crucial step in platelet activation [11,12,13,14]. Activated platelets express various integrins in their active conformation. Interactions of several platelet integrins (αIIbβ3, αvβ3, α2β1, α5β1, and α6β1) with their ligands (mainly fibrinogen, vitronectin, collagen, fibronectin, laminin) mediate platelet attachment to the vessel wall [13,14]. Upon platelet activation, different regulatory factors and secondary mediators, such as fibrinogen, vWF, adenosine di- and triphosphate (ADP/ATP), and serotonin, stored in intracellular alpha (α) and dense (δ) granules, are released, which then enhance prothrombotic events and stimulate the recruitment of circulating platelets to the site of injury. After platelet accumulation at the site of vascular injury, the second wave of this hemostatic process is mediated by the blood coagulation pathway, which generates thrombin from prothrombin through the extrinsic and intrinsic pathways [15]. Thrombin converts the soluble fibrinogen to fibrin, enhancing platelet activation and aggregation responses. In addition, activated platelets expose phosphatidylserine (PS) on their surface, which then facilities the recruitment of coagulation factor complexes that stimulate generation of thrombin in other vascular cells [15]. If occurring in diseased vessels, such as atherosclerotic arteries, the same events lead to vessel occlusion, which then can result in life-threatening pathological conditions, such as myocardial infarction or ischemic stroke. 

Platelet adhesion, activation, and aggregation are thus critical events in hemostasis and thrombosis and are tightly regulated by platelet glycoproteins, integrins and many other signaling receptors. Below, we discuss the effects of N-glycosylation on the function of the main platelet receptors and regulatory mechanisms controlling platelet adhesion and activation. 

### 2.1. Integrins

Loss or gain of N-glycan sites or contents may modify integrin expression and conformation, which consequently influence cell adhesion, migration, spreading, and the coagulation cascade. Integrins show much variation in the number and distribution of N-glycosylation sites. Studies by Cai et al. highlighted the importance of N-glycosylation on β3 integrin ligand binding and conformational modifications [16]. By site-directed mutagenesis and structure-guided analysis in HEK293 cells, the authors showed that the N-glycan site (N) 320 at the headpiece and leg domain of the integrin β3 interface positively regulates αIIbβ3 integrin activation [16]. In addition to this glycan site, β3-N559 at the β3-I-EGF3 and αIIb-calf-1 domain interface, and the β3-N654 at the β3-β-tail and αIIb-calf-2 domain interface positively regulate the activation of both αIIbβ3 and αVβ3 integrins [16]. Deletion of the β3-N371 glycan, located near the β3 hybrid and I-EGF3 interface, or β3-N452 in the I-EGF1 domain could enhance β3 integrin activation [16]. Interestingly, N-glycosylation at the βI domain of β1 subunit (β1-N343) negatively regulates fibronectin (FN) binding and α5β1 integrin activation [16]. Taken together, these data suggest that N-glycans may either positively or negatively modify ligand binding and integrin activation. N-glycosylation sites have also been found on human platelet α2 and α6 integrins [17], but their physiological role has not been investigated.

### 2.2. Glycoprotein VI 

Glycoprotein VI (GPVI) is one of the major collagen receptors in platelets, but it also interacts with other extracellular matrix molecules, such as laminin and fibrin [18,19,20,21,22]. GPVI signals through an immunoreceptor tyrosine-based activation motif (ITAM) and regulates diverse physiological processes in platelets including adhesion, activation, aggregation, and procoagulant activity [23,24]. In stably transfected DAMI megakaryocyte-like cell line, GPVI amino acid substitutions at position N92 or N94 resulted in a 90% or greater decrease in cell adhesion to the collagen-related peptide (CRP) and a 65% to 70% decrease to collagen [25]. These mutations do not affect protein expression or subcellular localization of GPVI, indicating that N-linked glycosylation of GPVI contributes to maximal adhesion to collagen or CRP [25]. Circulating platelets have stable levels of GPVI on their surfaces, but GPVI can undergo rapid proteolytic cleavage, generating soluble GPVI in the blood plasma. The shedding process is induced by GPVI ligands, such as collagen, fibrin, and venom toxins, or by elevated shear stress and active Factor Xa (FXa) [26]. GPVI can also be cleaved by disintegrin and metalloproteases ADAM10 and ADAM17 [26,27]. ADAM10 contains several potential N-glycosylation sites (N267, N278, N439, and N551) [28]. Mutation of N267, N439, and N551 did not completely abolish enzymatic activity of the ADAM10; however, reduced levels were found [28]. These results suggest that N-glycosylation can regulate ADAM10 function, resistance to proteolysis, and maintenance of the enzymatic activity.

### 2.3. C-Type Lectin-Like Receptor 2

The C-type lectin-like receptor 2 (CLEC-2) has been identified as an important platelet (hem)ITAM receptor, implicated in diverse platelet functions, although the natural ligands of this platelet receptor are still under investigation [29]. In platelet lysates, CLEC-2 is detected in two molecular masses, which are differently glycosylated. A specific glycosylation site is present on N134 [30], but its physiological role in platelet activation has not been further investigated. 

### 2.4. GPIb-IX-V Complex

The glycoprotein (GP) Ib-IX-V complex mediates vWF-dependent platelet adhesion at sites of blood vessel injury [13,31]. Molecular defects of this receptor complex account for the Bernard–Soulier syndrome (BSS). BSS variants show a decrease of GPIbα size and expression on the platelet surface [32]. Often, the receptor complex associates normally but displays defective processing of its N-linked glycans and abnormal O-glycosylation of GPIbα [33]. Confocal immunofluorescence microscopy reveals that mutant complexes mainly accumulate in the ER, whereas only a small proportion of the residual protein is successfully transported to the platelet surface [33]. In 2003, Hoffmeister et al. showed a major role of GPIbα in the clearance of cooled platelets [34]. Cold storage-induced clustering of GPIbα on the surface of murine platelets leads to the recognition of GPIbα by the αMβ2 integrin of macrophages [34]. Later, these researchers showed that exposed N-acetyl-D-glucosamine (GlcNAc) residues, present on N-linked GPIbα glycans, caused αMβ2 integrin recognition [35]. 

Another subunit of the complex, glycoprotein V (GPV), contains a single transmembrane domain, a short cytoplasmic domain, and a large extracellular domain with 8 potential N-glycosylation sites [36]. Mouse and human glycoprotein IX (GPIX) share 71% identical amino acids with highly conserved cysteine residues and a putative N-linked glycosylation site as well, located at residue N44 [37,38,39]. However, the physiological role of GPIX N-glycosylation has not been investigated in platelets.

### 2.5. G-Protein-Coupled Receptors

Thrombus growth requires mediators such as ADP, thromboxane A2 (TxA2), and thrombin, which act through G-protein-coupled receptors (GPCR). Platelet activation through GPCRs involves three major signaling pathways, initiated by the activation of Gα_q_, Gα_13_, and Gα_i_ proteins [40,41]. Platelet activation by ADP is mediated by the Gα_q_-coupled P2Y_1_ and the Gα_i_-coupled P2Y_12_ receptor, resulting in the inhibition of activated adenylyl cyclase [42,43,44]. Platelets from P2Y_1_-deficient mice do not undergo the usually observed shape change in response to ADP, and ADP-induced aggregation is severely impaired [45,46]. Moreover, mildly increased bleeding times and a relative resistance to ADP-induced thromboembolism has been observed for mice carrying a genetic deletion for the P2Y_1_ receptor [45,46]. Platelets from P2Y_12_-deficient mice have a normal shape change but impaired aggregation in response to ADP and prolonged bleeding time, and they form smaller and unstable thrombi [47,48]. The P2Y_12_ receptor contains two N-glycosylation sites in its extracellular N-terminus. Interestingly, N-glycosylation is dispensable for surface expression and ligand binding activity of the receptor, although non-glycosylated P2Y_12_ receptors are defective in the P2Y_12_-mediated inhibition of adenylyl cyclase activity, demonstrating the role of N-linked glycans in receptor signaling [49]. 

Protease-activated receptor (PAR) is also a member of GPCR receptors (GPCRs), playing a major role in thrombin-mediated platelet responses [50,51,52]. Activation of platelets by thrombin is essentially mediated by PARs, which couple to Gα_q_ and Gα_12_/G_13_ proteins [50,51]. Of the four PAR receptors, human platelets express PAR1 and PAR4, whereas mouse platelets express PAR3 and PAR4. PAR1 and PAR3 contain a hirudin-like domain, which has a high-affinity thrombin binding site. This interaction enables thrombin to bind and activate PAR receptors, resulting in cleavage [52]. The second extracellular loop (ECL2) of the PAR1 receptor is critical for specific ligand interactions and agonist recognition. PAR1 and ECL2 also contain two N-glycosylation sites, while the PAR2 isoform contains a single N-linked glycosylation site [53]. It has been shown that N-glycosylation of PAR1 at the surface of ECL2 influences ligand docking interactions that enhance the stabilization of the activated receptor complex and activation of G protein signaling [53].

## 3. Regulation of Calcium Homeostasis by N-Glycosylation

Calcium (Ca^2+^) homeostasis plays a crucial role in the regulation of platelet shape change, adhesion, activation, platelet granule release, and thrombus growth. Most platelet-activating receptors act through stimulation of phospholipase C (PLC) isoforms, catalyzing the hydrolysis of phosphatidyl 4,5-biphosphate (PIP_2_) into diacylglycerol (DAG) and inositol 1,4,5-triphosphate (IP_3_) [54,55]. These second messengers control receptor and store-operated Ca^2+^ entry (ROCE and SOCE). IP_3_ induces the depletion of the ER Ca^2+^ stores through the IP_3_-receptor (IP_3_R), which are integral membrane proteins of the ER, while DAG regulates Ca^2+^ entry through protein kinase C and activation of DAG-sensitive Ca^2+^ channels [55]. The receptor-induced Ca^2+^ entry mechanisms are mediated by ATP-gated P2X_1_ channel and DAG-regulated transient receptor potential channel 6 (TRPC6) [55,56].

P2X_1_ channel function has been well described in immune cells and platelets and contributes to thrombosis [57,58]. Drugs targeting the P2X_1_ receptor function have been proposed for the development of anti-thrombotic agents [42,43,57]. The role of N-glycans for P2X_1_ assembly, surface expression, and ligand recognition was analyzed using site-directed mutagenesis in *Xenopus* oocytes [59]. In this study, two out of the four naturally occurring N-glycans were sufficient for functional expression of P2X_1_ receptors at the cell surface, and residue N210 contributed to ATP potency [59]. 

Rat P2X_1_ contains five putative N-glycosylation sites which are all located in the ectodomain [59]. The N242 residue is absent in humans but not in the rat [59], indicating species-specific glycosylation of P2X_1_ in mammalians. Endo H treatment removes glycosylation chains, thereby reducing the molecular weight of P2X_1_. This is further reduced by PNGase treatment, indicating that P2X_1_ is glycosylated in the ER lumen and further in the Golgi [60]. Interestingly, mutations of residues N153, N184, and N242 did not affect ATP-mediated activation of P2X_1_ [60]. Residue F185 has been proposed to be involved in ATP binding, and N184 is located in this consensus motif [60]. N153, N184, and N242 are also glycosylated in human platelets, but their physiological function has not been investigated [17].

TRPC6 is highly expressed in both mouse and human smooth muscle cells, megakaryocytes, and platelets [61,62,63], and its activation is strongly dependent on PLC- and phospholipase D (PLD)-mediated DAG production [55]. By using site-directed mutagenesis in HEK293 cells, Dietrich et al. showed that elimination of the distal glycosylation site at N561 residue converted the tightly regulated TRPC6 into a constitutively active channel [64]. 

SOCE is the major Ca^2+^ entry route in activated platelets [54,55,65]. ER-resident Ca^2+^ is depleted to the cytoplasm, leading to activation of the stromal interaction molecule 1 (STIM1), which is the principal regulator of SOCE in platelets [54,55,66]; activated STIM1 is subsequently translocated from the ER to the plasma membrane-resident Orai1 channel [54,65,67]. Consequently, cytosolic Ca^2+^ levels are increased, mediating several physiological processes, including degranulation, integrin activation, and platelet aggregation [54,55,65]. The EF-hand domain of STIM1 is located in the ER lumen, and can be glycosylated at residues N131 and N171 [68]. It has been proposed that STIM1 N-glycans may modify the Ca^2+^ binding affinity of the molecule, as well as membrane stability and oligomerization with Orai1 [68]. In line with this notion, site-directed mutagenesis of the indicated N-glycosylation sites on STIM1 resulted in abolished SOCE in mammalian cells [68].

## 4. Regulation of Energy Metabolism by N-glycosylation

Platelet shape change and aggregation require a high concentration of ATP, which is stored and released by δ-granules upon platelet activation [15]. Platelets also possess the molecular machinery necessary to generate ATP through glycolysis and oxidative phosphorylation (OXPHOS) of glucose [69,70]. Platelets express several transporters of glucose and also can take up and oxidize fatty acids and glutamine as an alternative source of ATP generation [69,70]. Glucose transporters (GLUTs) are encoded by the solute carrier 2a (*Slc2a*) genes, transporting glucose through the plasma membranes of megakaryocytes and platelets [71,72]. Depending on the actual blood glucose concentrations, expression levels of GLUTs on the cell surface are either increased or reduced to maintain the basal glucose levels within the cells. Recently, a reduced platelet count has been detected in *Slc2a1^–/–^/Slc2a3^–/–^* double knockout mice with a combination of impaired pro-platelet formation and increased clearance of circulating platelets [73]. Interestingly, glucose uptake was completely blocked in *Slc2a1^–/–^/Slc2a3^–/–^* platelets, but mitochondria were able to take up alternative substrates to maintain energy metabolism. When mitochondrial function was further inhibited, *Slc2a1^–/–^/Slc2a3^–/–^* mice became severely thrombocytopenic, highlighting the important role of mitochondria in platelet production when glycolysis is severely impaired [73]. 

N-glycosylation regulates the binding affinity of GLUT1 to glucose and hence glucose uptake and transport [74,75,76,77]. Recently, quantitative glycoproteomics data and pulse-labeling experiments showed that the N45 residue of GLUT1 is N-glycosylated by the STT3B complex [78]. GLUT1 is hypo-glycosylated in lymphocytes, isolated from X-linked immunodeficiency with magnesium defect (XMEN) patients, and this (hypoglycosylation/reduced glycosylation of GLUT1) was also observed in *Magt1^−/−^Tusc3^−/−^* and *Stt3b^−/−^* HEK293 cell lines, indicating an important regulatory role of MAGT1 in N-glycosylation of GLUT1 during glucose uptake [78,79]. It is important to note that the glycosylation defects in XMEN lymphocytes are due to the absence of TUSC3 gene expression, the alternative compensatory mechanism of MAGT1 function [78]. More recently, Matsuda–Lennikov et al. also showed that MAGT1 function is partly interchangeable with TUSC3, but each protein has a different tissue distribution in humans [80]. The blood plasma of XMEN patients contains hypo-glycosylated haptoglobin, hemoglobin, and immunoglobulin heavy chain, which are regulatory elements of iron metabolism [80]. Therefore, such defects in N-glycosylation may also alter ATP transport and energy metabolism.

It has also been shown that STIM1-mediated SOCE and also calcineurin regulate the levels of GLUT1 and GLUT3, thereby influencing the efficiency of glycolysis [81]. Indeed, impaired Ca^2+^ influx through SOCE strongly inhibited the mRNA expression of glycolytic and mitochondrial enzymes in STIM1/2-deficient T lymphocytes [81]. In megakaryocytes and platelets, further investigation is necessary to understand the role of TUSC3, MAGT1, STIMs, and SOCE in the regulation of energy metabolic pathways by N-glycosylation and protein expression of GLUTs.

## 5. Regulation of The Coagulation Pathways by N-Glycosylation

α-Granules are the most abundant granules in platelets; their contents include adhesive glycoproteins, fibrinogen, vWF, coagulation factors, and granule membrane-specific proteins such as P-selectin [15,82]. Intracellular Ca^2+^ mobilization and granule release are essential steps to increase the procoagulant activity of platelets and to modulate the coagulation cascade [82]. Therefore, the modification of N-glycan composition on the platelet surface might be involved in the regulation of hemostasis and the coagulation cascade. 

P-selectin has two molecular sizes in platelets, and the high molecular weight form can be digested by peptide N-glycosidase F. The N-glycosylation inhibitor tunicamycin can suppress the surface expression of P-selectin, indicating a critical role of N-glycosylation in P-selectin trafficking [83]. Thrombin-stimulated platelets do expose P-selectin on the platelet surface and induce the phosphorylation of serine/threonine kinase 1 (AKT), but tunicamycin can inhibit this signaling event, suggesting that AKT signaling is involved in the tunicamycin-mediated inhibition of P-selectin expression [83].

Antithrombin III is a member of the serine protease inhibitor family. It is involved in the regulation of blood coagulation by inactivating several serine proteases and plasma factors, including thrombin, factor IXa, Xa, XIa, and XIIa [84]. Heparin enhances antithrombin III function; therefore antithrombin III deficiency or reduced heparin binding are involved in prothrombotic disorders [85]. Antithrombin III contains several putative N-glycosylation sites, namely N128, N167, N187, and N224 [86,87]. In antithrombin III deficiency, it has not been shown whether altered N-glycosylation may influence the heparin-binding of this serine protease inhibitor [87].

Histidine-rich glycoprotein (HRG) is mainly produced in the liver. It is involved in the regulation of immunity, coagulation, and angiogenesis and interacts with several plasma proteins and receptors in the blood. High levels of HRG are associated with symptoms of various cardiovascular disorders, while congenital deficiency of HRG results in thrombophilia in humans [88,89]. Three N-glycosylation sites have been confirmed in HRG, namely at N63, N125, and N344. A common human polymorphism has been described as well, creating a new glycosylation site at N202 [87]. However, so far, no reports have been published about the glycosylation status of HRG in the context of thrombotic disease or hemostasis. 

Kininogen or Fitzgerald factor (KNG) is mainly synthesized in the liver and exists in two molecular weights. It is expected to be highly glycosylated, based on the large N- and O-glycan structures, as the observed protein mass is more than 40 kDa higher than the mass calculated from the amino acid sequence [87,90]. The intact form of KNG is a cysteine proteinase inhibitor and has a major role in blood coagulation and inflammatory responses [91]. Depending on the physiological status of the blood, it can be cleaved into six different subchains which have diverse functions in the body [91]. KNG has four N-linked glycosylation sites at residues of N48, N169, N205, and N294 [87]. However, the effect of N-glycans on KNG function has not been investigated. 

Coagulation factor VII (FVII) has two N-glycosylation sites (N145 and N322). In transiently transfected COS-7 cells, mutations of these sites reduce the release of FVII to the medium and induce partial protein degradation [92,93]. These observations suggest an important role of N-glycans in the intracellular stability of the protein.

Coagulation factor XI is a disulfide-linked homodimer zymogen. Its deficiency causes bleeding diathesis in humans, referred to as hemophilia C [94]. N-glycosylation sites have been identified in both heavy (N72, N108, N145) and light chains (N432, N473) [95].

vWF is a key component of the coagulation cascade, acting as a bridging protein between the coagulant factors and platelet GPIb receptor, thereby mediating platelet adhesion at sites of vascular injury. Glycomics analysis of vWF showed about 100 distinct N-glycan structures [96]. Site-directed mutagenesis studies showed that individual glycans N99 (D1 domain), N857 (D’ domain), N2400 (B1 domain), and N2790 (CK domain) are critical for vWF synthesis and expression [97]. vWF becomes conformationally unfolded by shear stress in the circulation when it is cleaved by disintegrin and metalloprotease with thrombospondin type I repeats-13 (ADAMTS13) [98]. In a mutagenesis assay, N1574 glycan from isolated recombinant vWF-A2 domain increased the interaction of vWF with ADAMST13 and enhanced the susceptibility of vWF to ADAMTS13 proteolysis [99]. N-glycosylation contributes to vWF cleaving activity of ADAMTS13 by creating a conformational change of this protease optimal for interaction with vWF multimers [100]. However, once secreted, ADAMTS13 does not require N-glycans for its vWF cleaving activity [100].

## 6. Platelets in Congenital Disorders of N-Glycosylation

Congenital disorders of glycosylation (CDG) are a rapidly expanding group of genetic disorders due to defects in glycoprotein and glycolipid glycan assembly and attachment [101,102]. Since the first clinical report in 1980, approximately 130 different CDG have been identified. Most of them have an autosomal recessive inheritance. They can be divided into different subgroups: (I) defects in protein N-glycosylation (N-CDG), (II) defects in protein O-glycosylation, (III) combined protein glycosylation defects, (IV) lipid glycosylation defects, and (V) glycosylphosphatidylinositol anchor synthesis defects. Here, we review data on abnormal blood platelet number and function in N-CDG. It is important to note that platelet abnormalities, mainly thrombocytopenia, are mentioned only in a small group of N-CDG in the literature. On the other hand, in CDG patients with nonimmune hydrops fetalis, thrombocytopenia was reported in 53% (9/17) of patients, all in N-CDG patients, except one family with deficiency of subunit 6 of the conserved oligomeric Golgi complex (COG6)-CDG [103].

**ALG1-CDG** is due to deficiency of 1,4-mannosyltransferase 1, resulting mainly in a neurological syndrome, associated with variable involvement of other organs. Hematological abnormalities were found in 50% of these patients. These included thrombocytopenia (5/18 tested patients) besides general coagulation abnormalities and protein C and protein S deficiency [104].

**ALG8-CDG** patients are characterized by defective α-1,3-glucosyltransferase function. It is a severe disease since most patients died within the first year of life. Most patients showed prematurity, edema, dysmorphism, and gastrointestinal and cognitive impairment. In addition, coagulopathy and thrombocytopenia were noticed in the patients tested, but detailed platelet analyses have not been reported [105,106].

**MAGT1-CDG** results in X-linked immunodeficiency with Mg^2+^ defect, Epstein–Barr virus (EBV) infection, and neoplasia, called XMEN syndrome [107]. At the same time, it is also a defect in N-glycosylation since MAGT1 is an OST component [78]. Most patients developed chronic EBV-associated B cell lymphomas, caused by altered Mg^2+^ homeostasis in T-helper, cytotoxic T-lymphocytes, and natural killer (NK) cells [108,109]. Recently, Blommaert et al. reported new MAGT1-CDG patients with immunodeficiency and, in addition, developmental disability and hypoglycosylation [78]. MAGT1-mediated Mg^2+^ transport and its signaling has been proposed to act on the activation of PLC and subsequent intracellular Ca^2+^ release from the IP_3_-sensitive Ca^2+^ store [107]. In MAGT1-deficient cells, Mg^2+^ supplementation increased the free intracellular Mg^2+^ levels, most likely through Transient receptor potential cation channel subfamily M member 7 (TRPM7), TUSC3, or other alternative Mg^2+^ transport mechanisms [110,111]. Interestingly, Mg^2+^ supplementation effectively decreased EBV viremia in human patients after short-term treatment [112]; the effects of long-term treatment are not known. Recently, it has been shown that MAGT1-dependent glycosylation is sensitive to Mg^2+^ levels, and that reduced Mg^2+^ impairs immune cell function via the loss of specific glycoproteins, i.e., CD28 [80]. Most XMEN patients develop mild to moderate thrombocytopenia [112,113], although a detailed description of megakaryocyte and platelet functions have not been reported yet. *Magt^–/y^* mice have normal platelet count and size, although an altered ploidy of megakaryocytes was detected; but this mild defect did not influence platelet production [114]. Mg^2+^ is a known natural “platelet antagonist”, since elevated cytoplasmic Mg^2+^ concentrations ([Mg^2+^]_i_) inhibit platelet aggregation, cyclooxygenase activity, and TxA2 release [115]. Although many in vitro studies highlighted the effect of Mg^2+^ on hemostasis and coagulation, the regulatory roles of MAGT1 or other Mg^2+^ effectors and transporters in mouse models are still unknown.

**MPI-CDG** is a defect in mannose phosphate isomerase (MPI) that catalyzes the interconversion of fructose 6-phosphate and mannose 6-phosphate. It plays a critical role in maintaining the supply of D-mannose derivatives required for most glycosylation reactions [116]. MPI-CDG patients show a hepatic–intestinal presentation including thromboses besides life-threatening gastrointestinal bleeding. It is one of the very few treatable CDG with oral mannose supplements or liver transplantation [116]. Thrombocytopenia, as part of pancytopenia, has been reported in an adult, but no follow-up on platelet levels has been reported [117].

**PMM2-CDG** is a defect in phosphomannomutase 2 (PMM2) that catalyzes the isomerization of mannose 6-phosphate to mannose 1-phosphate. Subsequently, mannose-1-phosphate is converted into GDP-mannose, the source of mannose for the growing oligosaccharide chain. It is by far the most common N-glycosylation disorder and a multisystem disease with a highly variable phenotype. Characteristic clinical features are inverted nipples and peculiar fat pads [118]. These patients predominantly show a thrombotic tendency [118]. Platelets isolated from PMM2-CDG patients could aggregate spontaneously in platelet-rich plasma and at stirring conditions; the disappearance of single platelets was faster in whole blood. However, the platelet N-glycoproteome in PMM2-CDG patients showed unaltered N-glycosylation profiles of major platelet surface molecules, suggesting different mechanisms involved in hyperactivation of PMM2-CDG platelets [119]. Further studies are necessary to identify the molecular mechanisms, leading to thrombotic complications in these patients.

**SLC35A1-CDG** is a defect in the cytidine 5’-monophosphate (CMP)-sialic acid transporter that transfers sialic acid to the medial- and trans-Golgi. Patients show a neurological phenotype and bleeding diathesis associated with macrothrombocytopenia. Bone marrow aspirate of patients showed an increased megakaryocyte count with a predominance of immature forms. Evidence was found for a very short platelet life span as well [120,121,122]. A mouse model lacking specifically *Slc35a1* in megakaryocytes showed thrombocytopenia, decreased megakaryocyte maturation, and increased platelet clearance [123].

**STT3A-CDG** is a defect in a catalytic subunit of the OST complex. Patients show a neurological phenotype and exhibit severely decreased factor VIII (FVIII) secretion and lower plasma vWF level. Among seven patients, one had a mild thrombocytopenia, and one had normal platelet levels, and in the others there was no information on the platelet number [124]. 

## 7. Conclusions

A rather small number of studies have investigated the effects of defective N-glycosylation on platelet functions, and the pathophysiological role(s) of N-glycosylation in megakaryocyte/platelet functions remain(s) largely unknown. Studies using mouse models, e.g., with deficiency of OST complex components, are needed to clarify these issues. It would be interesting to investigate how far thrombocytopenia, coagulation defects, and thrombotic complications observed in CDG patients are a direct consequence of defective N-glycosylation in megakaryocytes/platelets.
